# Tunable diameter and spacing of double Ge quantum dots using highly-controllable spacers and selective oxidation of SiGe

**DOI:** 10.1038/s41598-019-47806-0

**Published:** 2019-08-05

**Authors:** Tsung-Lin Huang, Kang-Ping Peng, Ching-Lun Chen, Horng-Chih Lin, Tom George, Pei-Wen Li

**Affiliations:** Department of Electronics Engineering and Institute of Electronics, National Chiao Tung University, HsinChu, Taiwan (R.O.C.)

**Keywords:** Quantum information, Quantum dots

## Abstract

We report the novel tunability of the diameters and spacings of paired Ge double quantum dots (DQDs) using nano-spacer technology in combination with selective oxidation of Si_0.85_Ge_0.15_ at high temperature. Pairs of spherical-shaped Ge QDs were formed by the selective oxidation of poly-SiGe spacer islands at each sidewall corner of the nano-patterned Si_3_N_4_/poly-Si ridges. The diameters of the Ge spherical QDs are essentially determined by geometrical conditions (height, width, and length) of the nano-patterned spacer islands of poly-SiGe, which are tunable by adjusting the process times of deposition and etch back for poly-SiGe spacer layers in combination with the exposure dose of electron-beam lithography. Most importantly, the separations between the Ge DQDs are controllable by adjusting the widths of the poly-Si/Si_3_N_4_ ridges and the thermal oxidation times. Our self-organization and self-alignment approach achieved high symmetry within the Ge DQDs in terms of the individual QD diameters as well as the coupling barriers between the QDs and external electrodes in close proximity.

## Introduction

Quantum computing requires scalable quantum bits (qubits) that have to be controllably located in close proximity to each other as well as be individually addressable by external electrodes via tunable electron tunneling or capacitive coupling^1^. Several approaches have been proposed for the physical realization of qubits, including single photons^2^, trapped ions^3^, superconducting circuits^4^, single defects or atoms in diamond^5^ and in silicon matrices^6^, as well as semiconductor quantum dots (QDs)^7–10^. While encouraging achievements have been made using superconducting qubits operating at extremely low temperatures, recently, semiconductor QD qubits have emerged as the subject of intensive research not only for their great promise for high-temperature operation, but also for cost-effective fabrication using currently-existing semiconductor technology^11–13^.

Among the possible materials choices for semiconductor qubits, Si-based QDs are particularly attractive because complementary metal-oxide-semiconductor (CMOS) technology crucially enables the integration of Si-based QDs with Si CMOS electronics for qubit control, read-out and subsequent signal processing. To date, the operation of Si qubits has been validated only for very low temperature (<2 K) operation^13^. This is because that using current lithographic patterning technology, it is extremely difficult to produce sufficiently small-sized Si QDs (i.e. <5 nm; the excitonic Bohr radius is 4.9 nm for Si) that can be placed in close proximity to each other. Ge-based QDs are an exciting alternative for high-temperature qubit operation because of their larger exciton Bohr radius (~24.9 nm for Ge). Thus, Ge QDs have stronger quantum confinement as compared to Si QDs at comparable sizes. Another important benefit is that Ge QDs have stronger spin-orbital coupling than the corresponding Si QDs^14^, which is critical for electrically-driven spin qubits and spintronics applications. Therefore, it is important to establish reliable nano-fabrication schemes for generating size-tunable Ge QDs with controllable nearest-neighbor coupling.

In this paper, we report the experimental fabrication of a pair of double QD (DQD) system comprising of two spherical-shaped Ge QDs embedded within SiO_2_/Si_3_N_4_ matrices with tunable QD sizes and controllable QD spacings using a combination of CMOS fabrication technology and novel self-assembly growth techniques. This DQD system is formed by the thermal oxidation of nano-patterned poly-Si_0.85_Ge_0.15_ islands at the sidewalls of lithographically-patterned ridges of Si_3_N_4_/poly-Si. The diameter of individual Ge QDs is essentially determined by the geometrical conditions (thickness, width, and length) of the nano-patterned poly-Si_0.85_Ge_0.15_ spacer islands prior to thermal oxidation, whereas the interspace between the paired QDs is tunable by adjusting the width of the lithographically-patterned ridge in combination with the thermal oxidation time. Using our experimental approach, we have achieved high symmetry for our Ge DQDs in terms of both the geometrical sizes and shapes of Ge QDs as well as for the coupling barriers between the QDs and their external electrodes.

## Results

### Symmetrical double Ge quantum dots embedded within host matrices of SiO_2_/Si_3_N_4_

**Figure 1:**
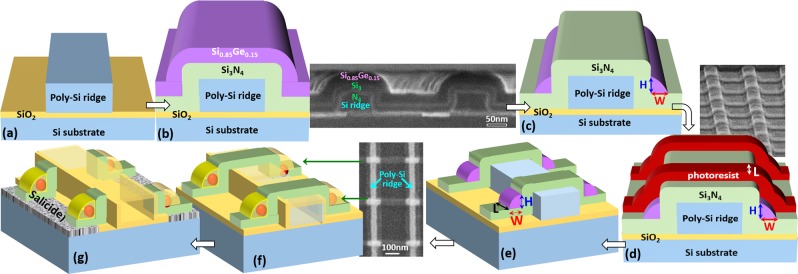
Detailed process flow diagrams showing the fabrication of double QDs embedded within SiO_2_/Si_3_N_4_ matrices via the thermal oxidation of nano-patterned SiGe spacer layers encapsulating Si_3_N_4_ layers that are deposited over ridges of poly-Si. (**a**) Lithographically-patterned, 20–75 nm-wide poly-Si ridges are formed over buffer layers of SiO_2_ on top of Si substrate. (**b**) Next, sequential deposition of 25 nm-thick Si_3_N_4_ and 30 nm-thick poly-Si_0.85_Ge_0.15_ layers conformally encapsulating the poly-Si ridges. Inset is the corresponding cross-sectional SEM micrograph. (**c**) Symmetrical spacer stripes of poly-Si_0.85_Ge_0.15_ are subsequently fabricated at each sidewall of the Si_3_N_4_/poly-Si ridges by a direct etch back process using SF_6_/C_4_F_8_ plasma. The widths and heights of the poly-Si_0.85_Ge_0.15_ spacer stripes are directly determined by the process times for deposition and etch back of poly-Si_0.85_Ge_0.15_ spacer layers. (**d**) Lithographically-patterning processes across the spacer stripes of poly-Si_0.85_Ge_0.15_ are conducted to define the lengths of the poly-SiGe spacer islands. Inset is the corresponding top-view SEM micrograph. (**e**) Symmetrical poly-Si_0.85_Ge_0.15_ spacer islands with widths (W)/heights (H)/lengths (L) of 20–30 nm/25–35 nm/30–40 nm are formed. Inset is the corresponding plan-view SEM micrograph. (**f**) Next, a pair of spherical Ge QDs is formed at each sidewall corner of the nano-patterned Si_3_N_4_/poly-Si ridge by thermal oxidation at 900 °C. (**g**) Finally, after a direct etch-back process in order to expose the Si surrounding the DQDs, self-aligned silicide (Salicide) external electrodes are fabricated by the deposition of either Ni or Ti, thermal annealing, and selective etching.

**Figure 2:**
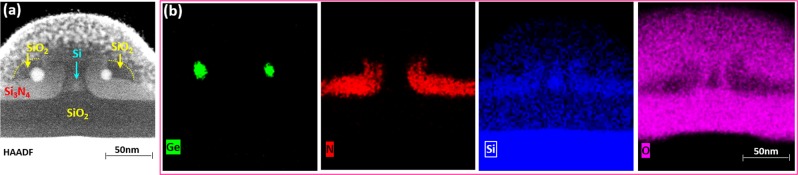
Transmission electron microscopy (TEM) observations following thermal oxidation of poly-SiGe spacer islands nano-patterned over Si_3_N_4_ layers deposited over poly-Si ridges. (**a**) STEM micrograph, (**b**) EDX maps of elemental Ge, N, Si, and O of the paired Ge QDs, each with diameter of 12 nm, formed at each sidewall of the poly-Si ridges.

Using the experimental fabrication procedure (Fig. 1) described in Method Section, we were able to fabricate a pair of Ge QDs simultaneously at each sidewall corner of nano-patterned Si_3_N_4_/Si ridges on top of the Si substrate, as shown by the scanning transmission electron microscopy (STEM) and energy dispersive *x*-ray spectroscopy (EDX) map micrographs in Fig. 2.

### Tunability of Ge quantum dot size

**Figure 3:**
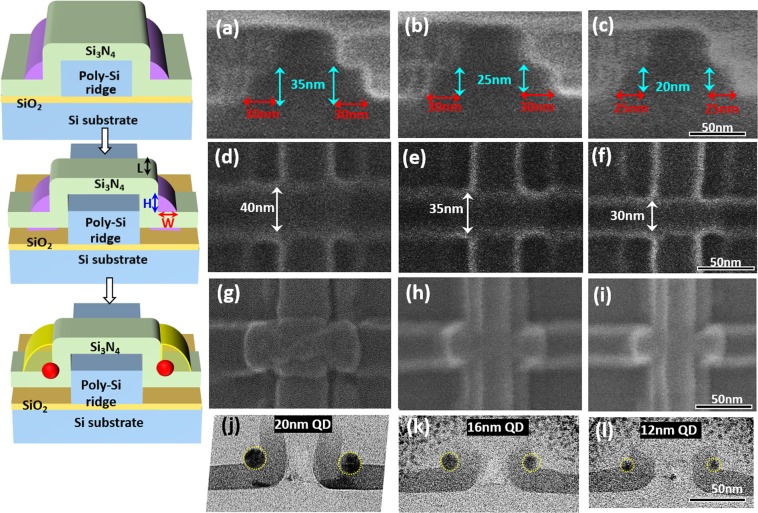
A pair of Ge DQDs simultaneously fabricated by the thermal oxidation of poly-SiGe spacer islands nanopatterned over Si_3_N_4_ layers encapsulating poly-Si ridges. 12 nm–20 nm Ge DQDs are produced by controlling the width (25–30 nm), height (20–35 nm), and length (30–40 nm) of the poly-SiGe spacer islands by varying the process times for poly-SiGe deposition and etch back in combination with the exposure dose of electron-beam lithography. Cross-sectional SEM micrographs of poly-SiGe spacer layers with Widths/Heights (W/H) of (**a**) 30 nm/35 nm, (**b**) 30 nm/25 nm, and (**c**) 25 nm/20 nm produced by using SF_6_/C_4_F_8_ plasma etch-back with process times of 7 sec, 9 sec, and 10 sec, respectively. Plan-view SEM micrographs of e-beam lithographically-patterned poly-SiGe spacer islands with lengths of (**d**) 40 nm, (**e**) 35 nm, and (**f**) 30 nm produced by exposure electron doses of 4.8 × 10^−16^ C, 5.4 × 10^−16^ C and 6.0 × 10^−16^ C, respectively. Following thermal oxidation at 900 °C for 10.5 min, plan-view SEM/cross-sectional TEM micrographs of (**g**,**j**) 20 nm, (**h**,**k**) 16 nm and (**i**,**l**) 12 nm diameter Ge QDs are produced by controlling the widths (25–30 nm), heights (25–35 nm), and lengths (30–40 nm) of the poly-SiGe spacer islands by varying the process times of poly-SiGe etch back and E-beam exposure. Inset on the left shows the corresponding structure schematic diagrams for clarity.

Most importantly, we successfully demonstrated precise control of the Ge QD diameter by controlling the process times for deposition and etch back for poly-SiGe spacer layers in combination with the exposure dose of electron-beam lithography for poly-SiGe spacer islands, thereby determining their width (W), height (H), and length (L), respectively. Thanks to the good conformity of low-pressure chemical vapor deposition (LPCVD) poly-SiGe spacer layers encapsulating the Si_3_N_4_/poly-Si nano-ridges as shown in the inset SEM micrograph of Fig. 1(b), the thickness of poly-SiGe spacer layers (i.e., the width of the poly-SiGe spacer islands) is essentially determined by their deposition time. The height of the poly-SiGe spacer islands is easily tailored by controlling the process times for poly-SiGe etch back, whereas the exposure dose of the second electron-beam lithography step essentially defines the lengths of the poly-SiGe spacer islands. We have conducted extensive experimental works in terms of the process times of etch back as well as electron-beam lithographic patterning for the production of size-tunable SiGe spacer islands. For a given deposition thickness of 30 nm for the SiGe spacer layers encapsulating the 25 nm-thick Si_3_N_4_/40 nm-thick poly-Si ridges, we have varied the width and height (W/H) of the SiGe spacer layers from 30 nm/35 nm to 25 nm/20 nm. This was achieved by increasing the process time of etch-back (using SF_6_/C_4_F_8_ plasma) from 7 to 10 seconds as shown in the cross-sectional SEM micrographs of Fig. 3(a–c). For varying the length of the SiGe spacer islands, which is essentially defined by the 2^nd^ electron-beam lithographic patterning step across the poly-Si nanoridges, we have additionally attempted 40 nm–30 nm lengths by increasing the exposure dose of electrons from 4.8 × 10^−16^ C to 6.0 × 10^−16^ C (i.e., by increasing the beam exposure time from 4.8 μs to 6.0 μs for a given electron-beam current of 100 pA). These results are shown in the plan-view SEM micrographs of Fig. 3(d–f). It is clearly seen from the plan-view SEM micrographs in Fig. 3(g,h) and in the cross-sectional TEM micrographs in Fig. 3(j–l), respectively, that pairs of spherical Ge DQDs with diameter of 12 nm, 16 nm, and 20 nm appear at each sidewall corner of the Si_3_N_4_/Si ridges following thermal oxidation (at 900 °C for 10.5 min) of poly-Si_0.85_Ge_0.15_ islands with widths/heights/lengths of 25 nm/20 nm/30 nm, 30 nm/25 nm/35 nm, and 30 nm/35 nm/40 nm, respectively.

### Controllability of inter-dot spacing

**Figure 4:**
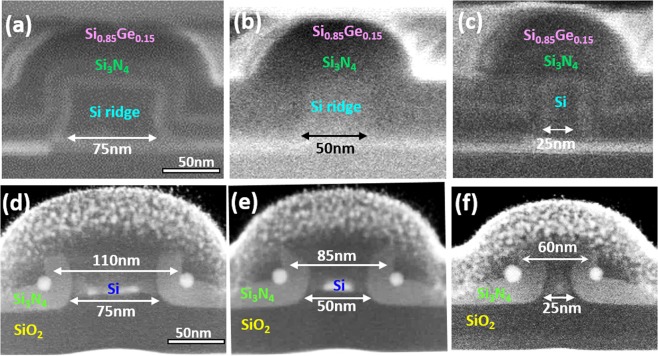
Cross-sectional SEM and TEM micrographs of 12 nm Ge DQDs formed by thermal oxidation at 900 °C for 10.5 min. The width of poly-Si ridges is decreased from (**a**) 75 nm, (**b**) 50 nm, and to (**c**) 25 nm, and the inter-dot spacing correspondingly decreases from (**d**) 110 nm, to (**e**) 85 nm, and to (**f**) 60 nm.

**Figure 5:**
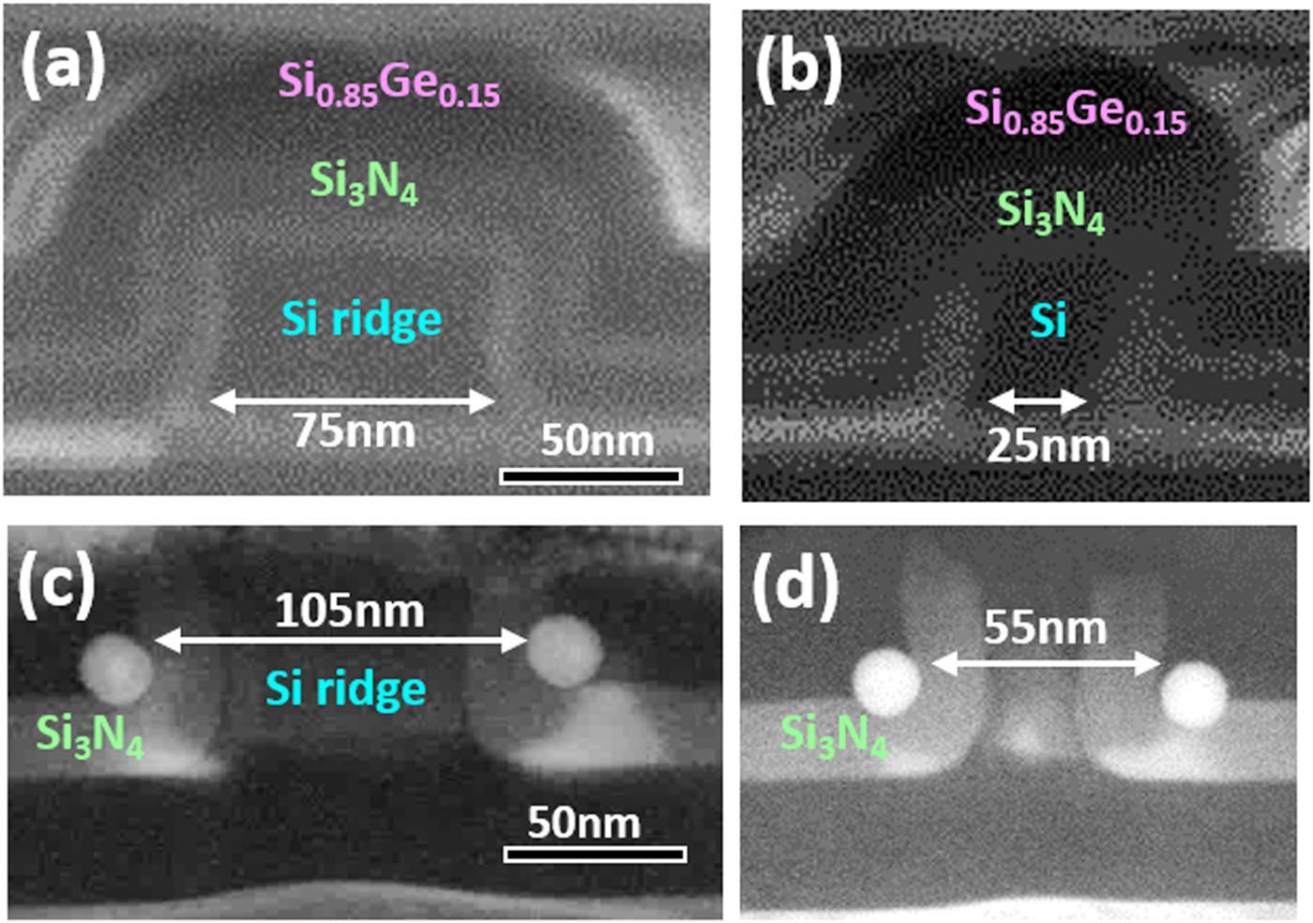
Cross-sectional SEM and TEM micrographs of larger, 20 nm Ge DQDs formed by thermal oxidation at 900 °C for 10.5 min. A decrease in the width of poly-Si ridges from (**a**) 75 nm to (**b**) 25 nm reduces the inter-dot spacing from (**d**) 105 nm to (**e**) 55 nm.

Based on our described fabrication approaches, several pairs of Ge DQDs were successfully, simultaneously produced at each sidewall corner of nano-patterned Si_3_N_4_/Si ridges. Figure 4 shows that following thermal oxidation at 900 °C for 10.5 min, the inter-dot spacing between double Ge QDs that are 12 nm in diameter was varied from 110 nm, 85 nm to 60 nm by decreasing the width of the lithographically-patterned poly-Si ridges from 75 nm, 50 nm to 25 nm, respectively. Further evidence for the tunablity of inter-dot spacing achieved by controlling the width of the nano-patterned Si_3_N_4_/poly-Si ridges is also shown for larger, 20 nm diameter Ge DQDs in Fig. 5.

**Figure 6:**
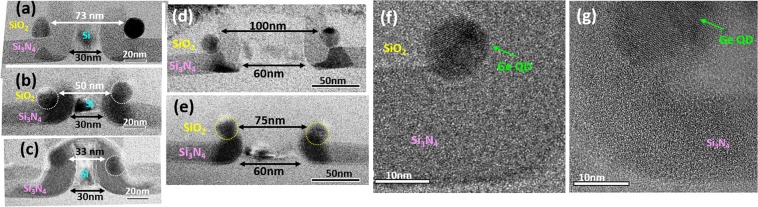
TEM micrographs of Ge DQDs formed at the sidewall corners of 30 nm-wide poly-Si ridges by thermal oxidation at 900 °C for (**a**) 10.5 min, (**b**) 27 min, and (**c**) 40 min. The depth of penetration of Ge QDs within the Si_3_N_4_ spacer layers is enhanced by increasing thermal oxidation time, leading to the reduction in the inter-dot spacing between the DQDs. The reduction in the inter-dot spacing with increasing thermal oxidation time is also evidenced for Ge DQDs in the cases of 60 nm-wide poly-Si nanoridges as shown for (**d**) 10.5 min and (**e**)27 min oxidation time. A longer oxidation process not only increases the penetration of Ge QDs through the Si_3_N_4_ layers, making the pair of QDs closer to each other, but also improves the crystallinity of the Ge QDs as shown in the HRTEM observations for (**f**) 10.5 min and (**g**) 27 min thermal oxidation at 900 °C.

Another important finding of note from Figs 2–5 regards the penetration of Ge QDs into the Si_3_N_4_ layer. Our previous papers have already reported that the depth of penetration of Ge QDs into Si_3_N_4_ is enhanced by increasing the thermal oxidation time^15–17^. In this work, we also observed that by increasing the thermal oxidation time from 10.5 min, through 27 min to 40 min, the separation between the double QDs is reduced by approximately 40 nm due to the significantly enhanced penetration of Ge QDs within Si_3_N_4_. These results are shown in Fig. 6 for the cases of the 30 nm-wide and 60 nm-wide poly-Si ridges. As shown in Fig. 6(a,d), 10.5 min of thermal oxidation at 900 °C produces a pair of Ge QDs that are located at each sidewall corner of the nano-patterned Si_3_N_4_/Si ridges. It is important to note that by increasing the duration of thermal oxidation at 900 °C to 27 min, the Ge DQDs have burrowed into the Si_3_N_4_ spacer layers for a depth of penetration of ~12 nm as shown in Fig. 6(b,e). Our experimental findings suggest that the spacing between the two Ge QDs is determined not only by the width of the nano-patterned Si_3_N_4_/poly-Si ridges, but also by the depth of penetration of the Ge QD into Si_3_N_4_ (dependent on the process time for thermal oxidation).

## Discussion

Detailed mechanisms for (1) the formation of spherical Ge QDs by thermal oxidation of poly-SiGe islands in close proximity to Si_3_N_4_ layers and (2) the penetration of Ge QDs through Si_3_N_4_ layers involve an exquisite, symbiotic interplay between Ge, Si, and O interstitials, have been described in detail in our previous publications^15–20^. In brief, the formation of Ge QDs by the thermal oxidation of the poly-SiGe islands located at the sidewall corners of the Si_3_N_4_/poly-Si ridges involves the preferential oxidation of Si and the Ge enrichment of the as-yet-unoxidized poly-SiGe regions. Ultimately, Ge crystallite clusters are formed by the progressive concentration of the Ge content within the remaining (unoxidized) poly-SiGe grains until the Si content is completely oxidized. With further oxidation, the Ge crystallite clusters can be made to penetrate the Si_3_N_4_ layers in close proximity due to Ge catalytically-enhancing the local decomposition and oxidation of Si_3_N_4_^15–17^. The migration of Ge QDs within Si_3_N_4_ involves a novel SiO_2_ formation-destruction mechanism^16–20^. Concurrent with the migration, the Ge crystallites grow in size by Ostwald Ripening culminating in complete coalescence, and resulting in a single, spherical Ge QD being formed at each sidewall corner of the Si_3_N_4_/poly-Si ridge.

The first important feature of our paired DQDs is the high symmetry observed both in Ge QD size and shapes, which is dependent on the dimensions of the nano-patterned poly-SiGe spacer islands at each sidewall corner of the Si_3_N_4_/poly-Si ridges. This is because the Ge content coalescing to form each spherical Ge QD during the selective oxidation of the poly-SiGe islands is exactly the same. The diameter of each spherical Ge QD is essentially determined by the geometrical sizes (width/height/length) of the poly-SiGe spacer islands. The width and height of the poly-SiGe spacer islands are tailored by controlling the process times of deposition and etch back for poly-SiGe spacer layers, whereas the second e-beam lithography step essentially defines the length of the poly-SiGe spacer islands.

The second important feature is the control of the spacing between the DQDs. The spacing between DQDs is primarily determined by the width of the nano-patterned ridge, and is also further reduced by the penetration of the Ge QDs within the Si_3_N_4_ overlayer. The exquisite control of the inter-Ge QD spacing lies in not only the conformal deposition of poly-SiGe spacer layers encapsulating the Si_3_N_4_/Si nanoridges, but also the controllable migration of the Ge QDs towards local sources of Si interstitials (emitted by either the Si_3_N_4_ or poly-Si layers)^16–18^. Increasing the process time of thermal oxidation not only facilitates Ge QD migration within the Si_3_N_4_ layer thus reducing the spacing between DQDs, but it also improves the crystallinity of the Ge QDs as shown in Fig. 4(e,f). The conformal spacer layers of Si_3_N_4_ over the poly-Si ridges are deliberately designed to be the initial, local source of Si interstitials to direct the Ge QD migration towards, reducing the inter-QD spacing.

The third important feature is that the resulting oxide layers, formed by the thermal oxidation of the poly-SiGe spacer islands, encapsulate the Ge QDs and serve as inherent tunneling barriers between the Ge QDs and external silicide electrodes in a self-organized manner. Once again, thanks to the processes of conformal spacer deposition, direct etch-back and thermal oxidation, symmetrical tunneling barriers between the DQDs and external electrodes could be simultaneously generated by our proposed fabrication processes. That is, following direct etch-back of the top oxide layers in order to expose the Si surface surrounding the DQDs and nanopatterned ridges (Fig. 1(f)), external electrodes could be subsequently formed by self-aligned refractory metal silicidation (also called salicidation) process (Fig. 1(g)).

**Figure 7:**
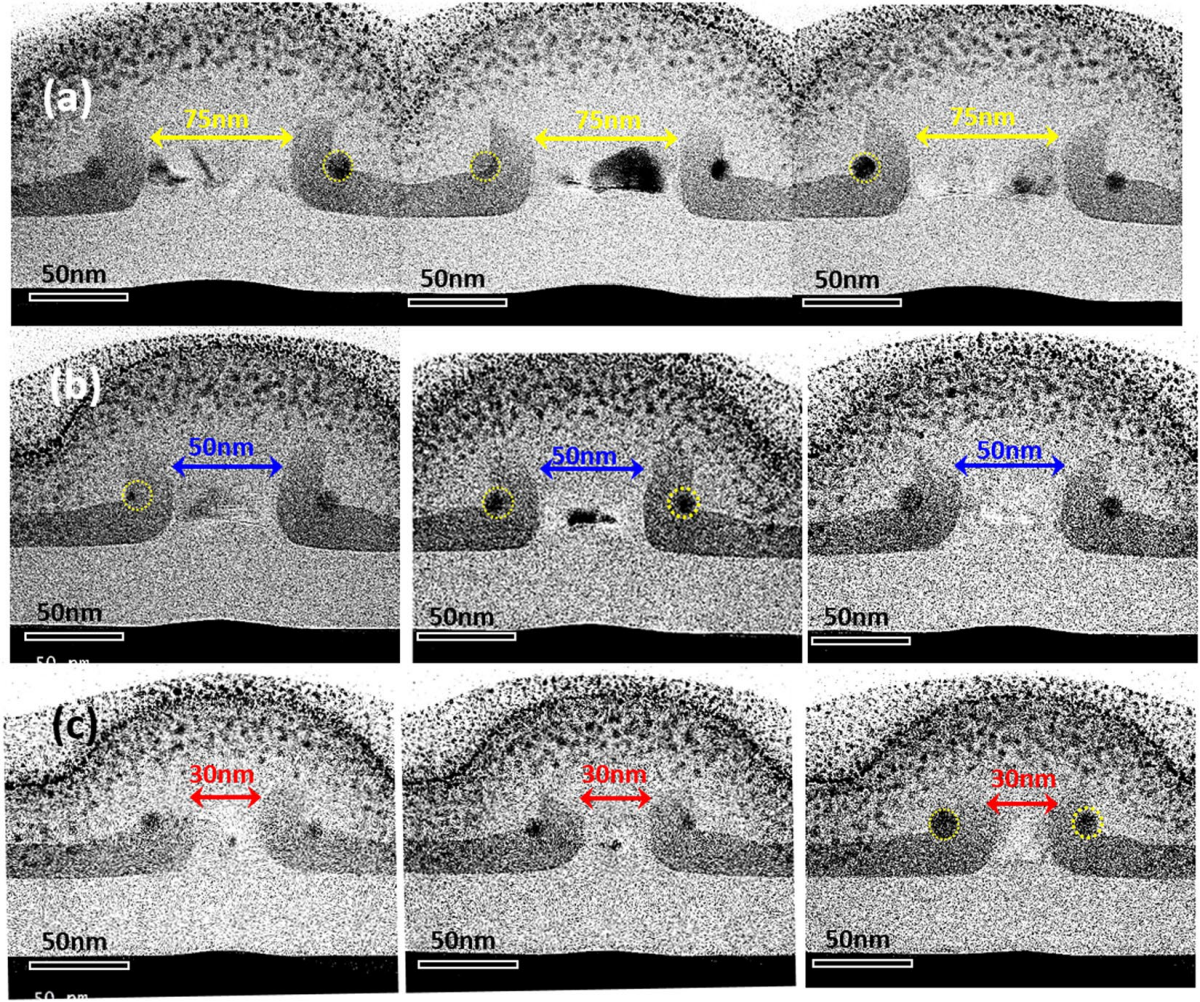
Extensive TEM observations of Ge QDs simultaneously fabricated at each sidewall corner of nano-patterned Si_3_N_4_/Si ridges with widths ranging from (**a**) 75 nm, (**b**) 50 nm and (**c**) 30 nm showing a high degree of symmetry, uniformity and reproducibility for the DQDs.

Based on our proposed approach, the tunability of the QD diameter and inter-dot spacing is achieved by a highly-controllable combination of nano-spacer fabrication, lithographic-patterning, and thermal oxidation of SiGe spacer islands. This combination, which includes the deposition and etch back of poly-SiGe spacer layers, lithographic patterning of SiGe spacer islands and Si ridges, and the thermal oxidation of SiGe spacer islands, employs standard fabrication processes in existing CMOS technology and therefore, by definition, is suitable for large-scale manufacturing. The uniformity of the resulting, fabricated Ge DQDs has been examined extensively using TEM/SEM observations. For further clarity, three TEM micrographs (Fig. 7) were included for each structure in order to demonstrate the high degree of symmetry, uniformity and reproducibility of our fabricated Ge DQDs under different combinations of process conditions.

**Figure 8:**
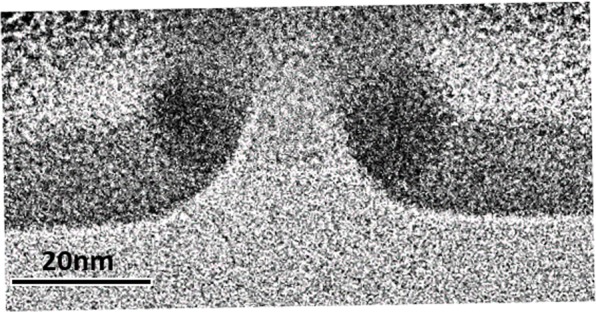
Cross-sectional TEM micrograph of symmetrical Ge DQDs with diameters as small as 12 nm and spacing between QDs as close as 13 nm formed by thermal oxidation at 900 °C for 27 min.

Using our controllable spacer and selective oxidation of poly-SiGe approach, a pair of symmetrical Ge DQDs with diameters as small as 12 nm and inter-dot spacing as close as 13 nm have been achieved (Fig. 8). Maurand *et al*. have reported the first Si-QD spin qubits implemented on a foundry-compatible Si CMOS platform using a 28 nm technology node with 64 nm pitch^11^. These reported Si-QD qubit devices, consisting of a two-gate pMOSFET (channel length of ~30 nm and inter-gate spacing of ~35 nm) within which, one gate defines a 30 nm-QD encoding a hole spin qubit and the other gate defines another 30 nm-QD for the qubit read-out. The authors demonstrated hole spin-qubit functionality with high fidelity at T = 10 mK. Our previous reports on the experimental fabrication of Ge-QD (with diameter of ~11 nm) single-hole transistors (SHTs) have already demonstrated clear Coulomb-blockade oscillatory current with peak-to-valley ratios (PVCR) > 100 and superior Coulomb stability at temperature as high as T = 77–140 K^21,22^. From the Coulomb-stability diagram, the extracted single-hole addition energy of 10–13 meV is a testament to the well-separated, discrete energy levels due to the strong quantum confinement effects for our 11 nm Ge QDs. By further downscaling the Ge QD sizes to 6 nm, we have demonstrated room-temperature operation of Ge-QD SHTs exhibiting clear Coulomb-blockade oscillatory current spectra with PVCR as high as 750^23^. Based on our previous accomplishments on Ge-QD SHTs, we believe that the operating temperature of qubits based on our Ge DQDs could be significantly increased to >100 K or even room temperature.

## Conclusions

We experimentally demonstrated the feasibility of paired, spherical-shaped Ge DQDs embedded within SiO_2_/Si_3_N_4_ matrices with tunable QD sizes and controllable inter-QD spacings using a CMOS nano-spacer fabrication technique in combination with selective oxidation of SiGe. The diameter of individual Ge QDs is essentially determined by the geometry (thickness, length, and width) of the poly-Si_0.85_Ge_0.15_ spacer islands prior to thermal oxidation. These dimensions are easily controlled by adjusting the process times of poly-SiGe layers deposition and their etch back. The inter-QD spacing is tunable by controlling both the width of the lithographically-patterned ridge as well as the thermal oxidation time. Using our experimental approach, we have achieved high symmetry for our Ge DQDs in terms of both the geometrical sizes and shapes of Ge QDs as well as the coupling barriers between the QDs and external electrodes. We envisage further scientific exploration of our Ge DQDs toward the ultimate goal of demonstrating advanced Ge-based QD qubit devices for practical applications.

## Methods

### Formation of self-organized, double Ge spherical quantum dots

The fabrication of paired Ge DQDs embedded within host matrices of SiO_2_/Si_3_N_4_ was initiated with the sequential deposition of bi-layers of 10 nm-thick SiO_2_ and 40 nm-thick poly-Si over Si substrates using low-pressure chemical vapor deposition (LPCVD). Poly-Si ridges of 25–75 nm in width were subsequently fabricated using a combination of electron-beam lithography and SF_6_/C_4_F_8_ plasma etching (Fig. 1(a)). Next, bi-layers of 25 nm-thick Si_3_N_4_ and 30 nm-thick poly-Si_0.85_Ge_0.15_ were sequentially deposited using LPCVD (Fig. 1(b)) in order to have conformal encapsulation over the poly-Si ridges. Following a direct etch-back process using SF_6_/C_4_F_8_ plasma (Fig. 1(c)), symmetrical spacer stripes of poly-Si_0.85_Ge_0.15_ with width/height (W/H) of 25–30 nm/20–35 nm were symmetrically produced by tuning the etch-back time at each sidewall of the Si_3_N_4_/poly-Si ridges. A second electron-beam lithography step in combination with SF_6_/C_4_F_8_ plasma etching was conducted across the poly-Si_0.85_Ge_0.15_ spacer stripes in order to define the length of 30–40 nm for poly-Si_0.85_Ge_0.15_ islands at each sidewall of the nano-patterned ridges (Fig. 1(d)). Finally, thermal oxidation at 900 °C for 10–27 min in an H_2_O ambient was performed to convert these poly-Si_0.85_Ge_0.15_ spacer islands to two spherical Ge QDs (Fig. 1(e)) at each sidewall corner of the ridges.

### Structural/chemical composition characterization

Structural properties and chemical composition of Ge DQDs embedded with SiO_2_/Si_3_N_4_ were assessed using cross-sectional high-resolution scanning transmission electron microscopy (STEM) and energy dispersive *x*-ray spectroscopy (EDX). The thickness of the TEM/EDX specimens were thinned to 60 nm by low-energy ion milling using focus ion beam (FIB). The Osiris & Talos TEM system operates at 200 kV and is equipped with a high-angle annular dark field (HAADF) detector. For EDX mapping, the camera length was 122 mm, and it took 20 min. to complete one mapping image.

## References

[1] Dennis E, Kitaev A, Landahl A, Preskill J (2002). Topological quantum memory. J. Math. Phys..

[2] Mi X (2018). A coherent spin-photon interface in silicon. Nature.

[3] Mehta KK (2016). Integrated optical addressing of an ion qubit. Nature Nanotechnology.

[4] Devoret MH, Schoelkopf RJ (2013). Superconducting circuits for quantum information: an outlook. Science.

[5] London P (2013). Detecting and polarizing nuclear spins with double resonance on a single electron spin. Phys. Rev. Lett..

[6] Tosi G (2017). *Silicon* quantum processor with robust long-distance qubit couplings. Nature Communications.

[7] Veldhorst M (2014). An addressable quantum dot qubit with fault-tolerant control-fidelity. Nature Nanotechnology.

[8] Yoneda J (2018). A quantum-dot spin qubit with coherence limited by charge noise and fidelity higher than 99.9. Nature Nanotechnology.

[9] Watson TF (2018). A programmable two-qubit quantum processor in silicon. Nature.

[10] Samkharadze N (2018). Strong spin-photon coupling in silicon. Science.

[11] Maurand R (2016). A CMOS silicon spin qubit. Nature Communications.

[12] Lo, C. C. & Morton, J. Silicon’s second act: Can this semiconductor workhorse take computing into the quantum era?, *IEEE Spectrum*, 37–43 (2014).

[13] Pillarisetty, R. *et al*. Qubit device integration using advanced semiconductor manufacturing process technology, in *IEDM Tech*. *Digest*, 133–136 (2018)

[14] Zhou Y (2011). Electrical spin injection and transport in germanium. Phys. Rev. B.

[15] Chien CY (2011). Nanoscale, catalytically enhanced local oxidation of silicon-containing layers by ‘burrowing’ Ge quantum dots. Nanotechnology.

[16] Chen KH, Wang CC, George T, Li PW (2014). The role of Si interstitials in the migration and growth of Ge nanocrystallites under thermal annealing in an oxidizing ambient. Nanoscale Res. Lett..

[17] George T, Li PW, Chen KH, Peng KP, Lai WT (2017). Symbiotic semiconductors: unusual and counter-intuitive Ge/Si/O interactions. J. Phys. D: Appl. Phys..

[18] Liao, P. H. *et al*. Self-organized gate stack of Ge nanosphere/SiO_2_/Si_1-*x*_Ge_*x*_ enables Ge-based monolithically-integrated electronics and photonics on Si platform. In *VLSI Tech. Dig*. Jun. 2018, pp. 157–158 (2018).

[19] Chen KH, Wang CC, George T, Li PW (2014). The pivotal role of SiO formation in the migration and Ostwald ripening of Ge quantum dots. Appl. Phys. Lett..

[20] Chen KH, Wang CC, Lai WT, George T, Li PW (2015). The pivotal role of oxygen interstitials in the dynamics of growth and movement of germanium nanocrystallites. CrystEngComm..

[21] Chen IH, Chen KH, Lai WT, Li PW (2012). Single germanium quantum-dot placement along with self-aligned electrodes for effective management of single charge tunneling. IEEE Trans. Electron. Dev..

[22] Chen IH, Lai WT, Li PW (2014). Realization of solid-state nanothermometer using Ge quantum-dot single-hole transistor in few-hole regime. Appl. Phys. Lett..

[23] Chen GL, Kuo DMT, Lai WT, Li PW (2007). Tunneling spectroscopy of a germanium quantum dot in single-hole transistors with self-aligned electrodes. Nanotechnology.

